# Ethnic and Gender Representation in German, Italian and Dutch School Textbooks

**DOI:** 10.1002/jcop.70063

**Published:** 2025-12-02

**Authors:** Astrid M. C. Jehle, Daudi van Veen, Marleen G. Groeneveld, Lotte D. van der Pol, Judi Mesman

**Affiliations:** ^1^ Leiden University College Leiden University The Hague the Netherlands; ^2^ Education and Child Studies Erasmus University Rotterdam Rotterdam the Netherlands

**Keywords:** ethnic representation, Europe, gender representation, occupational stereotypes, school textbooks

## Abstract

Female and/or ethnically minoritized textbook characters are often underrepresented and portrayed in ways that align with negative stereotypes. Previous studies have linked such stereotypes to educational underperformance and to educational choices that diverge from individual skills and preferences. In this study we aim to gain insights into the so‐called hidden curriculum; whether students observe a variety of ethnicity and gender among characters in their textbooks. In this cross‐national study, one mathematics and one language textbook from Germany, Italy and the Netherlands were analyzed. Ethnically minoritized characters, and specifically female ethnically minoritized characters were proportionally underrepresented. However, such characters were not underrepresented among main characters or those with an occupation. Ethnic and gender underrepresentation was more pronounced in the Italian textbooks than the Dutch and German textbooks. These preliminary results indicate a limited variety of female and/or ethnically minoritized textbook characters, which may reflect and strengthen educational inequalities.

School textbooks are a fundamental teaching and learning tool. They not only contribute to young people developing knowledge and skills according to the formal curriculum but can also function as a subtle socialization tool to shape young people's societal attitudes (Apple 2004). The latter is also referred to as the hidden curriculum, which includes values, stereotypes, and beliefs that are inadvertently or intentionally transmitted to students (Kentli 2009), such as that White people are the most skilled leaders (Ubaka et al. 2023), men are good at science (Miller et al. 2018) and young Black male college students are athletes (Higginbotham et al. 2022).

This paper examines how textbooks display diversity by acknowledging this hidden curriculum. It furthermore follows perspectives that emphasize how textbooks should promote respect for diversity and contribute to providing the knowledge and skills to appreciate cultural diversity and gender equality (UNESCO 2017). There are multiple ways to approach such a study, since textbook studies take place in a multidisciplinary field. Textbook studies comprise a diversity of topics and the research methods vary by discipline (Bock 2018). Currently, there is a change in the focus of textbook studies, from improvement of the content to analyses of the discourse in textbooks and the impact that textbooks have in the classroom (Bock 2018). The current paper is in line with this new focus and looks into the way characters in textbooks are represented.

## 1 Representation and Categorization

Representation may convey subtle socialization messages and negatively affect academic outcomes among students stereotyped as not being academically inclined (Lee 2014; McGill et al. 2012; Ullah and Skelton 2013). Studying numerical representation can reveal which groups are less visible (Biemmi 2015). Representation may also become evident from the role that characters play, because these roles convey messages about a character's place in society (Deckman et al. 2018). Numerical and role representation may vary by school subject. Studying subjects from the core curriculum, such as mathematics and language (European Commission 2020) may offer perspectives on the cues about ethnicity that young students obtain from their textbooks (Ferreira 2019; Ilett 2009; Koster et al. 2023; Van Veen et al. 2023). These two subjects are also considered gendered subjects: Mathematics is traditionally perceived to be a masculine subject, and language a feminine subject (Steffens and Jelenec 2011). This allows us to study subjects with different stereotypes associated with them, which, to date, only a limited number of studies have done (see e.g. Moser and Hannover 2014).

Furthermore, most textbook studies focus on one aspect of social categorization. Current scholars argue for an intersectional approach (Niehaus 2018), because gender and ethnicity of textbook characters can intersect (Crenshaw 1989). Intersectionality refers to the idea that stereotypes about a character assigned to one social category differ, depending on the combination with another social category (Shields 2008). Female characters are most often portrayed at home, as a spouse or caregiver, and only occasionally as a professional (Biemmi 2015). This varies however, depending on their ethnicity: Black female characters are less often portrayed as employed compared to White female characters (Ferreira 2019). This is relevant for studying inequalities in education, because from the lens of intersectionality, policies targeting unidimensional identities, such as gender, are insufficient to attain more diversity in education. Such an intersectional approach adds to our knowledge of who is in‐ and excluded. Therefore, the purpose of the present study is to examine ethnic and gender representation and their intersections in school textbooks by studying the proportions of (female) characters that are categorized as belonging to ethnically minoritized communities among textbook characters.

## 2 Representation in School Textbooks and Its Effects

Previous studies illustrate that people from ethnically minoritized communities are often numerically underrepresented in textbooks. For example, a U.S. study including undergraduate textbooks found that only 20% of pictures in introductory psychology textbooks showed characters with an ethnically minoritized background (Hogben and Waterman 1997), while this group corresponded with 27% of the population at that time (U.S. Census Bureau 2000b, 2000a). Likewise, a study on Dutch language textbooks revealed that ethnically minoritized characters are underrepresented relative to their proportion in the population; only 4% of the characters in pictures were ethnically minoritized characters (Koster et al. 2023), while 14% of the population had a migration background from the Global South (CBS 2022a). However, research comparing ethnic representation over time has found that while the proportion of ethnic minoritized characters is still limited, their representation in textbooks has increased (Hogben and Waterman 1997; Ilett 2009). Yet, numerical underrepresentation is a concern, because it may influence educational choices. Members of minoritized communities may experience reduced feelings of belonging in the academic or professional context, which in turn may lead them to choose another field of study or work (Bodamer 2020).

Studies on female characters show that the underrepresentation of women in textbooks is near‐universal (Blumberg 2008), examples range from 24.4% female characters in a Pakistani English language book (Islam and Asadullah 2018) to 46.5% in German language textbooks (Moser and Hannover 2014). Just as with ethnic representation, there are studies that show that the gap may be decreasing for gender representation as well. A study shows that female representation in German mathematics textbooks has increased over time, (Ott 2016). A study on German language textbooks has found an equal gender distribution among young characters (Moser and Hannover 2014). Given the indications that the gap in ethnic and gender representation may be decreasing, an examination of the intersection of these two identities is of interest.

Besides the quantitative representation, textbook studies illustrate that marginalized groups, such as ethnically minoritized communities and women, are portrayed in ways that align with negative stereotypes (Eigenberg and Min Park 2016; Sleeter and Grant 2017). Studies conducted in various countries suggest that characters that are categorized as belonging to ethnically minoritized communities may be characterized as primitive (König et al. 2021; Weiner 2016; Yamada 2011), or as having a low occupational status (Van Veen et al. 2023; Weninger and Williams 2005; Wigginton 2005). Many studies find that female characters are generally depicted in traditional gender roles, such as looking after the household and children (Blumberg 2008; Moser and Hannover 2014; Van de Rozenberg et al. 2023). Yet, when taking ethnicity into account, these results may vary. The intersection of ethnicity and gender in school textbooks has been explored in a small number of studies. These studies show that there are fewer female than male ethnically minoritized characters (Koster et al. 2023) and that Black female characters are less often described as doing a professional or intellectual activity than White female characters or Black male characters (Ferreira 2019). This may be problematic, because this points to a lack of role models in textbooks and it may ultimately deter minoritized students from pursuing their educational aspirations (Allen and Collisson 2020; Bodamer 2020). Furthermore, representation may influence majoritized students' behavior towards other groups if they come across stereotypes about these other groups in their textbooks. Because stereotypes contain a set of beliefs about a social group, when a person is categorized as a member of a social group, these beliefs create expectancies that guide the perception of and interactions with this person (Hamilton and Sherman 1994). For instance, adolescents often draw on stereotypes to justify social exclusion of peers (Mulvey et al. 2010).

In contrast, research on gender roles and career choices indicates that counter‐stereotypical role models may influence students' beliefs, and performance; role models motivate minoritized students to pursue their aspirations in counter‐stereotypical fields (Good et al. 2010; Olsson and Martiny 2018). Similarly, being exposed to role models from ethnically minoritized communities is related to positive psychosocial outcomes in ethnically minoritized youth. For example, simply reading about an African American role model reduced African American youths' negative self‐stereotyping (Rivera and Benitez 2016). A few studies find similar results for textbooks: Primary school children that used a more gender‐balanced textbook, showed more egalitarian attitudes towards stereotypically gendered activities (Karniol and Gal‐Disegni 2009). Therefore, these studies support the notion that textbooks should show a diverse representation of different types of people, to ensure that their ambitions are stimulated and that the readership is motivated to learn and grow (Bishop 1990). The above considerations beg the question to what extent and in which social contexts ethnic minoritized groups are represented in educational textbooks, not only in terms of numerical representation but also in terms of the social roles they occupy and the presence of potential stereotypes.

### 2.1 The European Context

The European context is particularly interesting for studying the role of ethnic representation in textbooks, because of its history of colonization and (invited) migration. We exploratively examine textbooks from the Netherlands, Germany and Italy: countries with similar migration histories. All three countries experienced an influx of migration in the 20th century. Migration waves started in the 1950s for the Netherlands and Germany. In the Netherlands they included migrant‐groups from former colonies. In Germany, the first waves included mostly ethnic Germans, but both the Netherlands and Germany saw increases in (recruited) labor migration in subsequent decades (Hansen 2003). The rise in migration, by refugees and labor migrants, in Italy started in the 1980s (Azzolini et al. 2019). Many people in these migration waves were invited to Germany, Italy, and the Netherlands, or arrived on their own initiative from countries and regions outside Europe, such as Morocco and the Caribbean. In other words, the ethnic diversity of these nations increased rapidly during the second half of the 20th century. Nowadays, one in four children in the EU were born outside the country in which they reside, or have parents who were born abroad (Orav 2023). Therefore, it is important to examine and describe the hidden curriculum that these children will be exposed to in their academic journeys (Appel et al. 2015; Mulvey et al. 2010; Thoman et al. 2013).

### 2.2 The Current Study

The purpose of the current study is to examine subtle messages regarding ethnicity and gender (and their intersections) that secondary school students observe in their textbooks. Therefore, we set out to study how ethnically minoritized characters, and specifically female ethnically minoritized characters, are represented in school textbooks. While most previous studies focus on either ethnicity or gender in textbooks, and on textbooks from one country or one subject, in the current study, data is compared between two subjects; mathematics and language, and among countries with a similar migration background: Germany, Italy, and the Netherlands.

Based on previous research, we hypothesize that ethnically minoritized characters, and specifically female ethnically minoritized characters are proportionally underrepresented among characters in school textbooks (H1). Extending previous research, we hypothesize that ethnically minoritized characters, and specifically female ethnically minoritized characters are proportionally underrepresented among main characters (H2). Based on previous research, we hypothesize that ethnically minoritized characters, and specifically female ethnically minoritized characters are proportionally underrepresented among characters with an occupation (H3), and that they have a lower occupational status than White characters (H4). Lastly, we exploratively look into possible differences among the proportional underrepresentation of ethnically minoritized characters, and specifically female ethnically minoritized characters in textbooks from Germany, Italy and the Netherlands (H5).

### 2.3 Methods

#### 2.3.1 Materials

##### 2.3.1.1 Textbooks and Textbook Selection

The sample of the present study comprises two types of school textbooks: A mathematics textbook, and a language textbook for learning the national language. Our sample consists of six textbooks in total: For each country, Germany, Italy, and the Netherlands, a mathematics and a language textbook for the age group of 12–13 years was analyzed. The Dutch textbooks were coded as part of a larger national study (Van de Rozenberg et al. 2023; Van Veen et al. 2023). The German and Italian textbooks were coded as part of a larger cross‐national study (Jehle et al. 2024). The textbooks were selected by the coders. They retrieved the most recent and most used mathematics and language textbooks from their birth country, see Table 1. These textbooks were published in the time period in 2013–2019. Because Germany and the Netherlands have separate educational tracks for this age group, but Italy does not, the textbooks that were selected for these two countries were used in the academic tracks. In Germany, textbooks are approved by the Federal State. In Italy, teachers choose textbooks, these textbooks can get licensed by the Ministry of Education. In the Netherlands, the government does not approve textbooks, publishers are required to adhere to learning targets in the textbooks. Neither the titles of the textbooks nor the names of the publishers are disclosed because of privacy reasons.

**Table 1:** Overview of textbooks selected for the current study.

Subject	Country	Year published	Selected and available textbooks
Mathematics	Germany	2013^a^	Textbook by the 3rd largest educational publisher. A total of three available textbooks, by two publishers.
	Italy	2017^a^	Textbook by the 2nd largest educational publisher. At least 10 available textbooks, by at least six publishers.
	The Netherlands	2019	Textbook by the largest educational publisher. A total of three available textbooks, by two publishers.
Language	Germany	2015^a^	Textbook by the 2nd largest educational publisher. A total of eight available textbooks, by three publishers.
	Italy	2017^a^	Textbook by the largest educational publisher. At least 15 available textbooks, by at least 11 publishers
	The Netherlands	2019	Textbook by the largest educational publisher. A total of three available textbooks, by three publishers.

^a^ This is to date the most recent edition.

#### 2.3.2 Coding and Procedure

While some scholars argue that it is problematic to apply categorical analysis to textbook characters, as this obscures their complexity (Quist 2013), we follow Spivak's approach of strategic essentialism (Spivak 2012) to identify attributes to study inequalities in textbooks. Strategic essentialism is a perspective explaining how we need categories, even if they do not reflect the actual diversity among group members, to encourage a shared identification when it is politically useful to have one (Spivak 1996). An extensive coding system was developed to code ethnicity and gender in textbooks. This coding system was described in a coding manual, that explained the variables, definitions and scores. Both the coding manual and the data from this study will be made available on OSF. The coding and collecting of the Dutch and cross‐national data were done separately but there where regular meetings between the authors to discuss the coding manual and data collection. All characters in text and images were coded, textbooks were analyzed from cover‐to‐cover, by one coder for each textbook.

The coder of each textbook was a native speaker, born in the respective country. Coders were undergraduate‐level students, assisting in exchange for course credits. There were two female and two male coders, they were part of the ethnically majorized group in their country. They were intensively trained in several training sessions to become familiar with the coding guide and the data. For the Dutch study, they had to complete a reliability set consisting of 20 pages and 15 characters from a mathematics and a language textbook in Dutch. For the German and Italian textbooks, this set consisted of approximately 40 pages and 15 characters from a mathematics and a language textbook in English. All coders participated in feedback sessions during the coding process. The coders identified all characters in the textbooks and coded whether they appeared in the text or an image, their ethnicity, gender, and role, including whether they were a main or side character and their occupation. They also provided a character summary with all relevant information. Additionally, a separate set of coders, unaware of the research goals and other variables, rated the names of the characters to determine ethnicity. This second group of coders were also native speakers; these were professionals with at least an undergraduate education. Among these coders were five female and two male coders, all part of the ethnically majoritized group in their country. As compensation, they were given a voucher valued at €20. This second group of coders only rated the ethnicity of the names; they did not code any other variables.

#### 2.3.3 Cultural Context

To understand the cultural context in which this study took place, three factors were examined: Ethnicity, gender, and nation. Regarding ethnicity, this study was done within a predominantly White context at a university in Western Europe. However, the authors of this paper formed an ethnically mixed group, including authors that identify as members of ethnically minoritized communities and others as White, with expertise in how gender and ethnicity‐related attitudes and beliefs (e.g., prejudice) are socialized. The expertise and various lived experiences in our team undoubtedly shaped our choices in the research process, since we are all driven to better understand how to best support youths' development. The coders were born in the respective country and were native language speakers. This enabled them to pick up on country‐specific cultural finesses such as descriptions of local ethnically minoritized communities or who is a main character. However, all coders were part of the ethnically majoritized community in their country and may have had stereotypical views on characters that are categorized as belonging to ethnic minoritized communities. During the coding discussion meetings, cultural differences in the data and equal attention to each group under study were discussed and noted in the data set (such as whether English names such as Mike refer to White or ethnically minoritized people, coders from each country were asked for their perspective).

Another relevant factor is the gender of the researchers. The authors and coders formed a group consisting of nine female and five male researchers. Working in a predominantly female team has given us the advantage that we are more aware of gender issues, some of us have lived experienced of such issues. Our mixed‐gender background and our lived experiences may have been formative to a focus on the female perspective and a drive to study the importance of gender equality in education. We are aware that these experiences do not make us immune to gender biases or to endorsing male‐centered values.

The last factor is the national perspective. The authors were based at the same university, located in the Netherlands. This could have led to a research perspective based on Dutch cultural beliefs and Dutch practices related to perspectives and practices pertaining to gender inequality and ethnic minoritized communities. However, one team member also received education in Germany, and is familiar with German cultural perspectives and practices on migration and gender equality. None of the authors has close ties to Italy, so the team relied on the coders native to Italy to provide insights on Italian national perspectives once issues arose. There were ample opportunities for the coders to provide input during the research process. During meetings we addressed several times how cultural perspectives may differ and invited team members to share their perspectives. Yet, as a team we may have been less able to relate findings to the Italian context, in comparison to our ability to do so within the Dutch and German context.

#### 2.3.4 Variables

##### 2.3.4.1 Ethnicity

Characters were included if their ethnicity (ethnically minoritized, White majoritized) could be approximated. For characters in images, the first set of coders categorized the ethnic appearance of the character. Characters were categorized as appearing to be White (e.g. pale skin, freckles) or ethnically minoritized (e.g. brown skin, Afro‐textured hair). If they appeared to be part of a specific ethnically minoritized group, this was noted. For example, characters with medium brown skin and Middle Eastern clothing were coded as being Middle Eastern, characters with medium brown to dark skin or afro‐textured hair were coded as being Black, and characters with dark straight hair and slanted eyes were coded as being Asian. Characters with an ambiguous ethnic appearance but who appeared to be a member of an ethnically minoritized group were only coded as being characters of an ethnically minoritized community. For the Dutch textbooks, two coders coded all the characters in images. A small number of ambiguous cases from the data set (characters that the coders found difficult to categorize into a specific ethnic group) were discussed to reach a consensus. For the German and Italian textbooks, one coder for each country rated the images in both the mathematics and language textbook. The inter‐rater reliability between the coders from the two countries was acceptable (Krippendorffs α = 0.68). All image ratings were checked by the first author by comparing the scores with the character summary. No adjustments were necessary.

Concerning the ethnicity of characters in the text, coders noted each name and registered whether ethnicity was mentioned (e.g. “Chinese exchange student”). When no information about the ethnicity of the character was mentioned, the name of the character was rated by the second group of coders. These coders assessed the origins of the name of the character. We chose this method, because previous studies showed how cues derived from names can influence performance assessment (Doornkamp et al. 2022). Each name was independently rated by two coders, who were both born and raised in the respective country. Firstly, coders were asked to indicate whether each name is associated with a person whose parents and grandparents were born in their country (Germany, Italy, or the Netherlands). Such characters were coded as White and local. Local refers to the country where the textbook was published. If not, coders indicated whether this name was associated with a person whose parents and grandparents were born in a country where members of dominant groups have White European ancestry and who do not belong to an ethnically minoritized group in this country. These countries were specified as a country in Europe, or Canada, the United States, Australia, or New Zealand. Such characters were coded as White but not local. For example, ‘Andreas’ was considered a White German name, and ‘Yasmin’ a name from the Global South. In cases where the two coders disagreed on the origin of a name, a third coder was consulted. This occurred for 24% of the German names, 9% of the Italian names, and 25% of the Dutch names. If the third coder did not agree with either of the first two coders, the origin of a character's name was deemed too ambiguous and excluded from the analysis. Coders were also able to indicate whether they were unsure about a name, if coders agreed about being unsure, the name was excluded from the analysis. 4% of the German, Dutch, and Italian names were eventually excluded from the analysis because of disagreement or ambiguity. When presumedly White characters with nonlocal names were excluded from the analyses, similar results were obtained. Therefore, White characters with local and nonlocal names were analyzed together.

Importantly, we want to note that we do not claim to know how the characters in the textbooks self‐identify. Instead, this coding procedure uses social cues regarding ethnicity to approximate what readers may infer regarding the group membership of the characters in question. Therefore, in our study the terms White and ethnically minoritized were used to discern between characters who are likely perceived as a) White or members from the majoritized groups in the countries of interest, or b) from characters who are likely perceived as having ancestry tracing to racialized groups from the Global South (i.e., Latin America, Africa, Asia, Oceania). We are aware that these terms are not in line with terms that are currently appropriate to describe ethnic diversity, because they imply clear differences between the two groups while the diversity within the groups is extensive (see CBS 2022b). Within the European context, groups who trace their ancestry to the Global South tend to face more marginalization than groups perceived as White (Moffitt et al. 2020), for this reason we focus on this distinction in the current study. We are also aware that there are groups with ancestry in Europe, such as Roma communities, that face racialization and prejudice in Europe. However, the representation of such characters in German, Dutch and Italian textbooks is very limited (see e.g. Spielhaus et al. 2020), for this reason they were not included in this study.

##### 2.3.4.2 Gender

Only characters for whom their gender (male or female) was discernible were coded. In the case of images, gender was assumed by whether their appearance aligned with cues stereotypically associated with femininity (e.g., long hair or skirts) or masculinity (e.g., having a beard). In the case of text, the gender of the character was assumed by their name, the pronouns referring to them, or a gender‐specific term. The probability of agreement among coders was high, 96%–100% for the German and Italian textbooks, 90%–93% for the Dutch textbooks.

We are aware that we have used binary gender categories, which is not in line with insights on the nonbinary nature of gender (Morgenroth et al. 2021). Yet, we chose to follow only clear cues consistent with gendered expectations (Maccoby 2000) because our goal was to look into stereotypes among textbook characters and because research has shown that even children with flexible gender expressions still categorize gender along the gender binary in the same manner as gender‐conforming children would (Glazier et al. 2020).

##### 2.3.4.3 Main Characters

In stories with one character, this character was always coded as the main character. When stories had multiple characters, the character from whose perspective the situation was being told or the character that was central to the situation as described was coded as the main character. When multiple characters contributed to the situation equally, they were all coded as main characters. In images, characters were coded as the main character when they were the center of attention in comparison to the other people around them. For example, in a mathematics book, Nina and Johanna want to go camping and start preparing. They are both the main character in this situation. An example in a language textbook are people at a concert, the performer is the main character, the spectator is a side character. The joint probability of agreement among coders for the German and Italian textbooks was acceptable (71%–91%), and high (89%–95%) for the Dutch textbooks.

##### 2.3.4.4 Occupational Roles

Coders registered whether a character had a professional paid occupation (e.g., journalist, engineer, baker). Additionally, they categorized the occupation according to the International Standard Classification of Occupations, ISCO‐08 (International Labor Organisation 2012). This classification was converted into the International Socioeconomic Index of Occupational Status, ISEI‐08 (Ganzeboom 2010), which represents the average level of education and the average income of characters with those occupations. The ISEI scale ranged from 10 points for kitchen helpers in food preparation to 89 points for medical doctors. For the Dutch textbooks, the inter‐coder reliability was 100%. For the German and Italian textbooks, occupational status was coded by the first author, no issues arose during the classification of the occupations in these textbooks.

#### 2.3.5 Data Analysis Plan

The hypotheses were tested separately for each country and school subject. This involved testing all hypotheses in two sets for each country: Characters from mathematics and language textbooks were analyzed separately. All analyses were performed in SPSS (IBM Corp. 2020). All results were tested against a significance level of 0.05. Confidence intervals of 95% were reported. We did not correct for multiple testing. Each hypothesis was considered a family and there were only two analyses, without overlap in sample, for each family of tests.

For our comparisons on numerical representation, we ran the following analyses: To test the hypothesis that ethnically minoritized characters were underrepresented, their proportion in the textbooks was compared to the proportion of the population for each country with ancestry tracing to racialized groups from the Global South. To test the hypothesis that female characters of color were underrepresented, their proportion in the textbooks was compared to the proportion of the female population in each country with ancestry tracing to racialized groups from the Global South. For these comparisons, binomial tests were conducted, and Cohen's *h* (Cohen 1988) was used to estimate the effect size.

For our comparisons on representation in roles, we ran the following analyses: To test the hypothesis that (female) ethnically minoritized main characters were underrepresented, the proportion of (female) ethnically minoritized main characters was compared to the proportion of (female) ethnically minoritized characters in the textbooks. To test the hypothesis that (female) ethnically minoritized characters were underrepresented among those with an occupation, the proportion of (female) ethnically minoritized characters among those with an occupation was compared to the total proportion of (female) ethnically minoritized characters in the textbooks. For these comparisons, binomial tests were conducted, and Cohen's *h* (Cohen 1988) was used to estimate the effect size. To test the hypothesis that characters that are categorized as belonging to ethnically minoritized communities had a lower occupational status than White characters, t‐tests were conducted. The types of occupations were listed descriptively, without running statistical analyses.

For our comparisons on cross‐national differences, we compared effect sizes: To test the hypothesis of cross‐national differences, Cohen's *h* (0.2 = small; 0.5 = medium; 0.8 = large) effect sizes were compared (Cohen 1988). To test for cross‐national differences in occupational status, Cohen's *d* (0.2 = small; 0.5 = medium; 0.8 = large) effect sizes were compared (Cohen 1988). The analysis plan was preregistered on the Open Science Framework.

### 2.4 Results

#### 2.4.1 Preliminary Statistics and Analyses

In 2021, Germany's population was 82.35 million people, of which 51% were female (DStatis 2023). Italy had a population of 59.24 million people (51% female; Istat 2022) and the Netherlands had a population of 17.48 million people (50% female; CBS 2022a). Germany, Italy, and the Netherlands do not collect information on people's self‐identified ethnic background. Instead, population statistics of governmental agencies about first and second generation migrants with a ancestry tracing to countries in the Global South provided an estimate of the size of the population that is likely to be perceived as ethnically minoritized (Balestra and Fleischer 2018). This population accounted for 13% of Germany's population (DStatis 2023), 5% of Italy's population^1^, and 14% of the Dutch population (CBS 2022a). Approximately half of the general population and half of the population with ancestry tracing to the Global South were female (CBS 2022a; DStatis 2023); see Table 2.

**Table 2:** Proportion of ethnically minoritized population and characters, main characters, characters with an occupation by country and textbook.

Country	Germany		Italy		Netherlands	
	All characters	Female characters	All characters	Female characters	All characters	Female characters
% of ethnically minoritized country population	13.14	6.47 (female)	5.37	2.47 (female)	14.00	6.98 (female)
% Minoritized characters						
Mathematics textbook	2.78*	0.46*	0*	0	4.56*	2.46*
Language textbook	11.11	5.05	1.32*	0.44*	7.80*	2.45*
% Minoritized main characters						
Mathematics textbook	1.14	0.57	0	0	4.26	2.33
Language textbook	6.82	2.27	1.90	0.63	7.45	1.86
% Minoritized characters among those with an occupation						
Mathematics textbook	17.65^a^	0	0	0	8.33	5.56
Language textbook	12.50	2.50	0	0	8.74	2.91

* Significant difference between the proportion of the population in each country that is (female) ethnically minoritized and the proportion of (female) ethnically minoritized characters in the textbook at *p* < 0.05.

^a^ Significant difference between the proportion of ethnically minoritized characters among those with an occupation and the overall proportion of ethnically minoritized characters in the textbook at *p* < 0.05.

Our data set consisted of 1,389 characters, of which 216 characters in a German mathematics textbook, 99 in a German language textbook, 112 in an Italian mathematics textbook, 228 in an Italian language textbook, 285 in a Dutch mathematics textbook, and 449 in a Dutch language textbook. In our study, White characters were compared to ethnically minoritized characters (i.e., with a minoritized background tracing to the Global South). We realize that our focus on characters with a minoritized background tracing to the Global South does not capture all people who may experience racialization in the Dutch, German, or Italian context. Previous research has shown there are only very few minoritized characters in school textbooks. Therefore, we hope our focus on one overarching category will provide us with a large enough sample size to draw conclusions.

The data set includes characters from both text and images. Only a few images, ranging from 0 to 11 per country, included ethnically minoritized characters. When characters in images were excluded from the analyses, similar results were obtained. Therefore, both the characters from text and images were analyzed together.

#### 2.4.2 Representation of (Female) Ethnically Minoritized Characters

The proportion of ethnically minoritized characters was compared to the proportion of the ethnically minoritized population in each country, see Table 2. Binomial analyses showed that compared to the ethnically minoritized population in Germany (13.14%), ethnically minoritized characters were underrepresented in the German mathematics textbook (2.78%, *N* = 6, CI [1.03%–5.95%], *p* < 0.005, *h* = 0.40), but not in the German language textbook (11.11%, *N* = 11, *p* = 0.33). Similarly, when looking specifically into female ethnically minoritized characters, it was found that, compared to the female ethnically minoritized population in Germany (6.47%), female ethnically minoritized characters were underrepresented in the German mathematics textbook (0.46%, *n* 2 = 1, CI [0.01%–2.55%], *p* < 0.005, *h* = 0.36), but not in the German language textbook (5.05%, *n* = 5, *p* = 0.36).

For Italian textbooks, binomial analyses showed that, as compared to the proportion of the Italian ethnically minoritized population (5.37%), ethnically minoritized characters were underrepresented in the Italian mathematics (0%, *N* = 0, CI [0%–3.24%], *p* = 0.01, *h* = 0.45) and language textbook (1.32%, *N* = 3, CI [0.27%–3.80%], *p* = 0.01, *h* = 0.22). As compared to the proportion of the female Italian ethnically minoritized population (2.47%), female ethnically minoritized characters were not underrepresented in the Italian mathematics textbook (0%. *n* = 0, *p* = 0.08), but they were underrepresented in the Italian language textbook (0.44%, *n* = 1, CI [0.017%–2.42%], *p* = 0.04, *h* = 0.18).

For Dutch textbooks, binomial analyses showed that, as compared to the population in the Netherlands (14.00%), ethnically minoritized characters were underrepresented in the Dutch mathematics textbook (4.56%, *N* = 13, CI [2.45%–7.67%], *p* < 0.005, *h* = 0.34) and in the Dutch language textbook (7.80%, *N* = 35, CI [5.49%–10.67%], *p* < 0.005, *h* = 0.20). Similarly, as compared to the female population in the Netherlands (6.98%), female ethnically minoritized characters were underrepresented in the Dutch mathematics textbook (2.46%, *n* = 7, CI [0.99%–4.99%], *p* < 0.005, *h* = 0.22), as well as in the Dutch language textbook (2.45%, *n* = 11, CI [1.23%–4.34%], *p* < 0.005, *h* = 0.22). When considering country differences, effect sizes *h* suggest that among the mathematics textbooks the underrepresentation of ethnically minoritized characters was larger in Italian than in German and Dutch textbooks. For female ethnically minoritized characters, effect sizes *h* suggest that the underrepresentation of ethnically minoritized characters was larger in German than in Dutch mathematics textbooks. No effect size was calculated for the Italian mathematics textbooks because the comparison of proportions was not significant. Among the language textbooks effect sizes *h* suggest that the underrepresentation of (female) ethnically minoritized characters were similar for Italian and Dutch textbooks. No effect size was calculated for the German language textbook because the comparison of proportions was not significant.

In sum, these results support our first hypothesis, that in most textbooks under study (female) ethnically minoritized characters are underrepresented as compared to the population in the respective country. For our fifth, exploratory, hypothesis, the results suggest that textbooks from Italy show a larger underrepresentation than those from Germany and the Netherlands.

#### 2.4.3 Representation Among Main Characters

The proportion of ethnically minoritized characters that were main characters was compared to the proportion of ethnically minoritized characters in each textbook to measure how integral the ethnically minoritized characters are to the storylines in the textbooks. The preliminary analysis indicated a very small number of ethnically minoritized characters that were main characters, which presents a challenge for detecting differences in proportions through statistical tests. The data indicates that the proportions of ethnically minoritized characters that were main characters was low across all textbooks and countries, see Table 2. An examination of the gender distribution within the type of textbook reveals that the proportion of female ethnically minoritized characters that were main characters in mathematics textbooks is approximately half of the proportion of all ethnically minoritized characters that were main characters. While the proportion of female ethnically minoritized characters that were main characters in language textbooks is closer to one‐third. However, none of the the statistical tests detected significant differences: In the German mathematics textbook, ethnically minoritized characters that were main characters (1.14%, *N* = 2, *p* = 0.14) were not underrepresented, neither were female ethnically minoritized characters that were main characters (0.57%, *n* = 1, *p* = 0.50). In the German language textbook, ethnically minoritized characters that were main characters (6.82%, *N* = 3, *p* = 0.25) were not underrepresented, neither were female ethnically minoritized characters that were main characters (2.27%, *n* = 1, *p* = 0.31). It was not possible to compare the proportions of main characters in the Italian mathematics textbook because there were no ethnically minoritized characters in this textbook (*N* = 0). In the Italian language textbook, ethnically minoritized characters that were main characters (1.90%, *n* = 3, *p* = 0.40) were not underrepresented, neither were female ethnically minoritized characters that were main characters (0.63%, *n* = 1, *p* = 0.50). In the Dutch mathematics textbook, ethnically minoritized characters that were main characters (4.25%, *N* = 11, *p* = 0.47) were not underrepresented, neither were ethnically minoritized characters that were female main characters (2.33%, *n* = 6, *p* = 0.50). In the Dutch language textbook, ethnically minoritized characters that were main characters (7.45%, *N* = 28, *p* = 0.44) were not underrepresented, neither were female ethnically minoritized characters that were main characters (1.86%, *n* = 7, *p* = 0.28).

In sum, these results do not support our second hypothesis, because (female) ethnically minoritized characters that are main characters are not underrepresented as compared to the proportion of ethnically minoritized characters in the respective textbook.

#### 2.4.4 Representation Among Characters With an Occupation

The proportion of ethnically minoritized characters among those with an occupation was compared to the proportion of ethnically minoritized characters for each country, see Table 2. The preliminary analysis indicated a limited number of ethnically minoritized characters among those with an occupation, which, similarly to analysis on main characters, presents a challenge for detecting differences in proportions through statistical tests (Sauro and Lewis 2005). In the German mathematics textbook, binomial analyses showed that among characters with an occupation, ethnically minoritized characters were overrepresented (17.65%, *N* = 3, CI [3.80%–43.43%], *p* =0.01, *h* = 0.53) compared to all ethnically minoritized characters in the German mathematics book (11.11%). No analyses were conducted on female characters, because among characters with an occupation, there were only male, no female ethnically minoritized characters. In the German language textbook, ethnically minoritized characters among those with an occupation were not underrepresented (12.50%, *N* = 5, *p* = 0.49), neither were female ethnically minoritized characters underrepresented (2.50%, *N* = 1, *p* = 0.35), compared to the proportion of the overall proportion of (female) ethnically minoritized characters. There were no ethnically minoritized characters in the Italian mathematics books and so we could not compare for ethnically minoritized characters among those with an occupation. In Dutch textbooks there was no underrepresentation among characters with an occupation: Ethnically minoritized characters were not underrepresented in the mathematics book (8.33%, *N* = 3, *p* = 0.25), female ethnically minoritized characters were not underrepresented either (5.56%, *n* = 1, *p* = 0.25). In the Dutch language book, ethnically minoritized characters were not underrepresented (8.74%, *N* = 9, *p* = 0.43), neither were female ethnically minoritized characters (2.91%, *n* = 3, *p* = 0.50). As most analyses are not significant, cross‐national comparisons through effect sizes are not applicable.

In sum, with the exception of characters in the German mathematics textbook, these results do not support our third hypothesis, because (female) ethnically minoritized characters among those characters with an occupation are not underrepresented as compared to the proportion of ethnically minoritized characters in the respective textbook.

#### 2.4.5 Representation and Occupational Status

The occupational status of characters that are categorized as belonging to ethnically minoritized communities among characters with an occupation was compared to the status of White characters. Our statistical analyses were focused solely on ethnic differences due to the small number of female ethnically minoritized characters in various textbooks: German textbooks contained only one female ethnically minoritized character among those with an occupation, Italian textbooks none, and Dutch textbooks five. For German textbooks, among characters with an occupation, the status of ethnically minoritized characters (*M* = 42.38, SD = 16.12) was lower than the status of White characters (*M* = 58.55, SD = 14.06; *t*(57) = 2.97, *p* < 0.005, *d* = 1.07). German textbooks featured ethnically minoritized characters that were authors, miners, athletes, and a soldier, while White characters were, among other occupations, described as scientists, artists, and teachers. For Italian textbooks, no comparisons were made, because there were no ethnically minoritized characters among those that had an occupation. For Dutch textbooks among characters with an occupation, the status of ethnically minoritized characters (*M* = 52.83, SD = 12.88) did not differ from the status of White characters (*M* = 58.27, SD = 13.53; *t*(128) = 1.33, *p* = 0.09). There was one outlier in the Dutch data: A White farmer. Running the analyses without this character did not change the result, so it was retained. Dutch textbooks featured ethnically minoritized characters that were athletes, a rapper or singer, a restaurant employee, an author, a furniture designer and a physical therapist. White characters were described in similar occupations, and additionally as business owners, managers, psychologists and scientists. Due to the variation by country in the degree to which ethnically minoritized characters are portrayed with an occupation, we deemed it not insightful to also run formal analyses comparing these representations between countries.

In sum, these results partly support our fourth hypothesis. Although we were not able to run the analyses for the Italian textbooks, we found that ethnically minoritized characters had a lower occupational status in German textbooks, but not in Dutch textbooks.

### 2.5 Discussion

Our goal was to examine subtle messages regarding ethnicity and gender, and their intersections, that secondary school students are exposed to in their textbooks. We examined the proportion of ethnically minoritized characters and female ethnically minoritized characters in mathematics and language textbooks from Germany, Italy and the Netherlands. Our findings demonstrate that (female) ethnically minoritized characters were underrepresented in most textbooks under study. (Female) ethnically minoritized characters were not underrepresented as main characters or with an occupation. Ethnic and gender underrepresentation was more pronounced in the Italian textbooks, as compared to the Dutch and German textbooks. These results show that the German, Italian, and Dutch textbooks under study do not reflect the ethnic and gender diversity in these countries and offers students a limited variety of characters in their textbooks.

In line with our expectations on the underrepresentation of ethnically minoritized characters, (female) ethnically minoritized characters were underrepresented in the mathematics textbooks of all three countries, compared to the proportion of ethnically minoritized people in the country's population. These findings are consistent with the results of previous studies on ethnic representation (Hogben and Waterman 1997; Sleeter and Grant 2017), on gender representation (Biemmi 2015; Moser and Hannover 2014), and on gender and ethnicity in textbooks (Ferreira 2019; Koster et al. 2023). It is notable that in the Italian mathematics book there were more than a hundred characters, none of them were categorized as belonging to ethnically minoritized communities. As for language textbooks, (female) ethnically minoritized characters were underrepresented in the Dutch and the Italian textbook, which was in line with our expectations. Contrary to our expectations, they were not underrepresented in the German language textbook. We will address these country differences further below. The current results show that there are very few models for students to teach them about the diversity of educational and career opportunities. This lack of role models also conveys an image of society to students of all ethnic backgrounds in which people that are a member of ethnically minoritized communities are not visible. Even through previous research has shown that both ethnic and gender representation in textbooks may have slightly increased over the last decades in some textbooks (Ilett 2009; Ott 2016), our study shows preliminary indications that a representation of female ethnically minoritized characters, as an intersection of these identities, may be limited. These results align with the intersectional invisibility model, which suggests that women of ethnically minoritized communities are often rendered culturally invisible through a lack of representation (and misrepresentation) in cultural products (Purdie‐Vaughns and Eibach 2008). In the case of textbooks, this intersectional invisibility may discourage female students of ethnically minoritized communities from pursuing their academic interests and talents (Bodamer 2020).

When looking into the roles that ethnically minoritized characters play in textbooks, we could not find evidence to support our hypothesis about main characters. The results of this study indicate that ethnically minoritized characters that were main characters were underrepresented as compared to the country's population, but they were not underrepresented among main characters in general. Still, the findings demonstrate that students seldomly get an opportunity to see ethnically minoritized characters in a central role in the situation and that such characters are given few opportunities to present a diversity of skills to students.

Additionally, we could not find evidence to support our hypotheses about representation among characters with an occupation. Our results indicate that ethnically minoritized characters are underrepresented as compared to the population in the countries under study, but not more as compared to how often ethnically minoritized characters appear in textbooks overall. The results of the current study demonstrate that students witness only very few examples of ethnically minoritized people, especially women, that have an occupation in the textbooks under study. However, our sample sizes for the occupational analyses were very small. Subsequently, differences for occupational representation may have been underestimated in the data analysis. Yet, it is noteworthy, that while there were a small number of examples of characters with an occupation in the Italian language textbook, none of these were ethnically minoritized characters. We will address these countries' findings further below. Our inspection of the variety of occupations of ethnically minoritized and White characters revealed a similar image. White characters are depicted in a wider variety of occupations, than ethnically minoritized characters, making the former more visible in the occupational context. In the textbooks under study, this offers White students a wider range of identification targets than it does for ethnically minoritized students.

Contrary to our hypothesis, the analysis revealed an overrepresentation of ethnically minoritized characters among those with an occupation in the German mathematics textbook. However, this textbook does not offer students a more diverse image of occupations: There were only male ethnically minoritized characters in the German mathematics textbook, and overall, the occupations of ethnically minoritized characters were of a lower status than those of White characters in the German textbooks. As long as ethnically minoritized characters only partake in the lower status occupations, this may reinforce the image of an ethnically homogeneous workplace for the top‐tier occupations (Howard et al. 2011; Lu and Li 2021). Also, seeing role models in lower‐status occupations may negatively influence the career ambitions of adolescents ethnically minoritized communities (Allen and Collisson 2020).

Exploratively, we examined country differences in representation of ethnically minoritized characters. For most country comparisons in the current study, we found that the Italian textbooks showed a larger underrepresentation of ethnically minoritized charactersas compared to the German and Dutch textbooks, for both the mathematics and the language textbook. This finding is in line with previous research arguing that the Italian curriculum is geared towards an ethnically homogeneous image of Italians where White Italians are centered (Coppola and Sabelli 2012; Kaliska 2022). Possibly, between‐country differences in (inter)national educational policies and initiatives offer an explanation for the country differences between Germany, Italy and the Netherlands (Faas 2013). At the time of writing of this article there were educational policies at work on ethnic diversity and inclusive education both in Germany and the Netherlands, but not in Italy (European Commission, & European Education and Culture Executive Agency 2023).

Furthermore, we found that underrepresentation of ethnically minoritized characters was smaller in the Dutch than in the German mathematics textbook. Contrary to Germany and other countries, in the Netherlands ‘Realistic Mathematics Education’ was integrated, which intends to make learning mathematics meaningful by using real‐life examples (Vos 2007), such as pictures of characters performing a calculation for a practical task. This approach may invite textbooks authors to include a slightly more diverse range of characters in Dutch as compared to the German mathematics textbooks.

Another finding that stands out from the results reported earlier is that there was no underrepresentation of ethnically minoritized characters in the German language textbook. This is in line with previous research concluding that the representation of ethnic diversity has increased in German language textbooks (Ilett 2009). Possibly this increase has evolved in the last decade. Culturally, this would be in line with the growing awareness within Germany and the German education system of being a multiethnic country (Bender‐Szymanski 2010). At the time of writing of this article, there were several governmental initiatives such as collaborations with a school textbook publisher on a prize for diversity‐awareness in schools, hands‐on guidelines for inclusive education, and recruiting teachers from ethnic minorities (Antidiskriminierungsstelle des Bundes 2019, 2024; European Commission, & European Education and Culture Executive Agency 2023). It is plausible that these initiatives have contributed to a more balanced ethnic and gender representation in German language textbooks.

#### 2.5.1 Limitations and Recommendations

The findings of this study must be considered in light of some limitations, to which we have added recommendations. Firstly, our study is based on a small sample, consisting of one textbook for each subject in each of three countries. As such, the results should be considered as preliminary. For some of the analyses, the number of characters in the comparison was very low and there is a risk of a sampling error. The current results present an indication of patterns of underrepresentation within textbooks for core subjects and within and between countries. Future research including a larger body of textbooks and textbooks from more countries is necessary to provide more insight into the generalizability of our findings and the extent of the underrepresentation of different ethnicities and genders in textbooks. For future research, we suggest including textbooks from other European countries, such as Sweden, that has an ambitious gender equality programme for schools (Umhauser‐Henning 2015) or Portugal, which is, at the time of writing this paper, implementing a national plan to fight structural inequalities concerning racism (European Commission, & European Education and Culture Executive Agency 2023).

Secondly, our estimation of the population with ancestry tracing to the Global South in Italy is most likely on the conservative side. Because the Italian National Institute of Statistics does not register the generational status of residents (Istat 2020; also see endnote 1), we had to make an estimation of second‐generation Italians with a background in the Global South based on a limited definition, and on data including only a subset of countries of ancestry. We anticipate that the actual number of ethnically minoritized residents will be higher than the current data reveals. Because the difference between the proportion of the Italian population with ancestry tracing to the Global South and the proportion of ethnically minoritized characters was significant, an even larger population proportion would not have changed the statistical conclusions. It may mean that people with ancestry tracing to the Global South are even more underrepresented in Italian textbooks than described in this study.

Lastly, incorporating more classroom materials in the study may yield additional insights on both cross‐country as well as teacher‐specific differences in ethnic and gender representation in education. Additional classroom materials include online resources connected to the textbook, as well as movie clips or quizzes and tests introduced by individual teachers, and increasingly, AI‐generated educational content (Denny et al. 2023). These materials convey socialization messages, just like textbooks. Research suggests that additional classroom materials lack diversity (Silver 2022). Similarly, AI‐generated content can be biased in how it portrays women (Fang et al. 2024) and ethnically minoritized groups (Fang et al. 2024; Yang 2025). Because textbooks are only one part of all classroom materials, it would be worthwhile to gain a more complete image of the ethnic and gender representation that adolescents perceive in the classroom environment by studying whether additional materials add to or average out messages on diversity representation. Previous research also suggests that teachers differ in the degree to which they use additional classroom materials that show diversity in ethnicity and gender (Silver 2022). It would be worthwhile to study cross‐teacher differences to understand more about the influence that teachers have on the lack of diversity representation in classroom materials.

#### 2.5.2 Conclusion

Results of the current study show a lack of variety in ethnic and gender representation among textbook characters in most of the textbooks in our study. It furthermore shows us that the textbooks in the current study do not reflect the ethnic and gender diversity in Germany, Italy and the Netherlands. Overall, our findings point to what can be described as a hidden curriculum in school textbooks. As previous research has demonstrated, there are negative effects of underrepresentation on educational choices and performance. Based on the patterns we uncovered in our study, it may merit for textbook publishers to offer a more diverse range of textbook characters, to improve equality in education.

## Ethics Statement

There are no human participants in this study and informed consent is therefore not applicable.

## Conflicts of Interest

The authors declare no conflicts of interest.

## Data Availability

The data that support the findings of this study are openly available in OSF at https://osf.io/tu3hx/overview?view_only=31d77c49f25f4b0c8335b05dea2ebc21.

## Endnotes

1 The Italian Institute of Statistics does not collect information on the generation of immigrants (Favazza and Pia 2006; Istat 2020). Instead, we used statistics on the number of foreign residents with a background from the Global South (Istat 2021b) and an estimation of naturalized Italians (Istat 2021a, 2021c) with a background from the Global South.

2
*N* refers to the total sample, e.g. ethnically minoritized characters in a mathematics book. *n* refers to the subsample, e.g. female ethnically minoritized characters in the same mathematics book.
